# Challenges and barriers to optimal maternity care for recently migrated women - a mixed-method study in Norway

**DOI:** 10.1186/s12884-021-04131-7

**Published:** 2021-10-07

**Authors:** Sukhjeet Bains, Susanne Skråning, Johanne Sundby, Siri Vangen, Ingvil K. Sørbye, Benedikte V. Lindskog

**Affiliations:** 1 Department of Obstetrics and Gynecology, Norwegian Research Centre for Women’s Health, Oslo University Hospital, Rikshospitalet; PO box 4950 Nydalen, 0424 Oslo, Norway; 2 Department of Community Medicine and Global Health, Institute of Health and Society, University of Oslo, Oslo, Norway; 3 Department of labour, Welfare and Local Communites, Stovner District, City of Oslo, Oslo, Norway; 4 Institute of Clinical Medicine, Faculty of Medicine, University of Oslo, Oslo, Norway; 5 Department of Social Work, Child Welfare and Social Policy, Oslo Metropolitan University, Oslo, Norway

**Keywords:** Migrant, Maternity, Antenatal, Norway, Barriers, Migration, Vulnerability, Qualitative, Questionnaire

## Abstract

**Background:**

Migrant women are at increased risk for complications related to  pregnancy and childbirth, possibly due to inadequate access and utilisation of healthcare. Recently migrated women are considered a vulnerable group who may experience challenges in adapting to a new country. We aimed to identify challenges and barriers recently migrated women face in accessing and utilising maternity healthcare services.

**Methods:**

In the mixed-method MiPreg-study, we included recently migrated (≤ five years) pregnant women born in low- or middle-income countries and healthcare personnel. First, we conducted 20 in-depth interviews with migrant women at Maternal and Child Health Centres (MCHC) and seven in-depth interviews with midwives working at either the hospital or the MCHCs in Oslo. Afterwards, we triangulated our findings with 401 face-to-face questionnaires post-partum at hospitals among migrant women. The data were thematically analysed by grouping codes after careful consideration and consensus between the researchers.

**Results:**

Four main themes of challenges and barriers faced by the migrant women were identified: (1) Navigating the healthcare system, (2) Language, (3) Psychosocial and structural factors, and (4) Expectations of care. Within the four themes we identified a range of individual and structural challenges, such as limited knowledge about available healthcare services, unmet needs for interpreter use, limited social support and conflicting recommendations for pregnancy-related care. The majority of migrant women (83.6%) initiated antenatal care in the first trimester. Several of the challenges were associated with vulnerabilities not directly related to maternal health.

**Conclusion:**

A combination of individual, structural and institutional barriers hinder recently migrated women in achieving optimal maternal healthcare. Suggested strategies to address the challenges include improved provision of information about healthcare structure to migrant women, increased use of interpreter services, appropriate psychosocial support and strengthening diversity- and intercultural competence training among healthcare personnel.

**Supplementary Information:**

The online version contains supplementary material available at 10.1186/s12884-021-04131-7.

## Background

Disparities in maternal health between migrants and host population in high-income countries remains a public health concern [1]. It is well established that migrant women have increased risk for several adverse outcomes during pregnancy and birth [2, 3]. The causes are complex. Both individual determinants, such as age, gender and genetics; and structural determinants, such as legal, political and socio-economic frameworks; play important roles in an individual’s health. Structural determinants can be especially important to a migrant’s health – both physical and mental – during the different stages of the migration and integration process [4]. A migration experience may also be associated with loss of social network and direct economic loss [5]. In addition, previous experience with fragmented healthcare and poor quality can affect trust in the health system of the host country.

Although migrant women are a heterogeneous group of people with huge variability in socioeconomic status and risk profiles, they share the experience of being new to a country. As such, recently migrated women are more likely to have a relative disadvantage compared to migrants with residence of more than 5 years, many of whom arrived as children and thus have greater language proficiency and familiarity with the health systems in host countries. Furthermore, women born in low- or middle-income countries constitute a vulnerable group with higher risk of receiving inadequate antenatal care, compared to the migrant women born in high-income countries [6].

Migrants may encounter barriers and challenges in utilizing the healthcare system due to language barriers, low health literacy, socio-economic difficulties, lack of psychosocial support, cultural beliefs, and low-transcultural proficiency of healthcare personnel [6–9]. ‘Barrier’ is understood as anything that restricts access, use or benefit from healthcare services, and a ‘challenge’ as a subjective experience of something that requires great effort to succeed and, in contrast to ´problem´, is an opportunity for growth [7]. Health literacy includes both personal and organisational health literacy [10]. The former focuses on the individual’s ability to find, understand and use information and healthcare services, whereas the latter focuses on the organisation’s ability to enable individuals to find, understand and use information and healthcare services [10].

Even though maternity care in Norway is generally considered to be of good quality, sub-optimal maternity care [11, 12] and barriers to health care access [13, 14] among migrants have been reported. Previous systematic reviews have explored the experiences of migrant women in accessing and utilising the maternal healthcare in host countries [15–17]. However, acculturalisation occurs over time and there is limited research on *recently* migrated women’s perceived barriers to optimal maternity care in Norway. Furthermore, quantitative research exploring the patterns of access and utilisation of maternal healthcare among recently migrated women is lacking.

This article is a part of the project “*The MiPreg Study: Closing the Gaps in Maternity Care to Migrant Women in Norway”*. The results will be used to pilot an intervention to fill gaps in maternal healthcare that decrease health disparities between migrants and host population. In order to develop efficient interventions, we need to map the current patterns of access and utilisation, and better understand the challenges this group face. Thus, the aim of this article was to identify challenges and barriers recently arrived migrant women face in accessing and utilising the maternity healthcare service in Norway. We strive for a comprehensive approach by utilising both qualitative and quantitative methods, as well as including the perspectives of both migrant women and midwives.

## Methods

### Study setting

This study is set in urban Oslo, the city with the largest population of migrants in Norway, with migrants currently accounting for 26% of the population [18]. The highest proportion of recent migrants born in low- or middle-income countries to Oslo in 2020, in descending order, were from Poland, Syria, Lithuania, Eritrea and the Philippines [18]. Norway has universal health coverage and compulsory healthcare insurance paid through taxes, that covers all care rendered in hospitals. Essential maternity healthcare before, during and after birth is free of charge for all residents in the country with a national identification number or temporary identification number, including refugees and asylum seekers yet to receive a residence permit. Persons without legal residence, such as undocumented migrants, are entitled to healthcare during pregnancy and birth, but while antenatal services are offered free of charge, they are financially responsible for expenses related to childbirth [19]. Pregnant women can choose to have their follow-up at their family doctor or a midwife at a Maternal and Child Health Centre (MCHC) [20]. The standard antenatal package includes eight consultations, including one routine ultrasound screening at around week 18. Almost all births in Norway are institutionalised and there are only public hospitals for delivery. After discharge from hospital the midwives at MCHC provide the post-partum follow-up.

### Inclusion criteria

We included pregnant migrant women in urban Oslo, with a length of stay ≤ 5 years in Norway and born in a low- or middle-income country. Thereafter, we used the Global Burden of Disease regional classification system, which is based on epidemiological similarity and geographic closeness, to classify women into different regions [21]. We included midwives with extensive experience in providing maternity care for migrant women from hospitals and MCHCs in urban Oslo. In the Norwegian maternity care system, midwives often provide the majority of antenatal and post-partum care and deliver most normal births. They often have a relational and social approach to migrant women and their families throughout the pregnancy. Due to these factors, we chose to include midwives as representatives for healthcare personnel.

### Study design and triangulation

The MiPreg project is a multidisciplinary, mixed-method project. It is organised into four parts, of which two are included in this article: quantitative part (structured questionnaire with migrant women) and qualitative part (in-depth interviews with migrants and healthcare personnel). We sought to triangulate our findings by technique, i.e., applying mixed-methods, with in-depth interviews from two different but interrelated groups – women and midwives, and a structured questionnaire among migrant women. Triangulation can be used to increase the validity in research as it combines different methods to answer a research question [22]. It enabled a different perspective to our study objective, and thus provided a more complete and comprehensive understanding about the subject of barriers and challenges migrant women face.

#### Quantitative part: structured questionnaire

In this part we applied a quantitative questionnaire, using a modified version of the Migrant Friendly Maternity Care Questionnaire (Supplementary file 1), that measures maternity care related factors in migrant populations [23]. To ensure accuracy and consistency of data collection the interviewers - three midwives and one physician, were trained and an interview guidebook was produced. In addition, the interviewers met regularly to discuss challenges and experiences. From January 2019 until February 2020 the interviewers at the two hospitals serving urban Oslo identified eligible pregnant women being admitted at the birth ward. The women were interviewed face-to-face in their own language of choice using an interpreter when needed, before discharge from the hospital. The mean completion time for the questionnaire was 44 min. A previously published article, provide detailed description on the methodology for the questionnaire-study [24].

#### Qualitative part: in-depth interviews with migrant women

In this part, two anthropologists experienced in qualitative methods conducted in-depth, semi-structured interviews with migrant women from March until December 2019. The interviews took place at three MCHC in Oslo with high proportions of migrants. We ensured variation in country of birth in the sampling process. Of the women recruited,15 were in their third trimester, and the remaining five had recently given birth. The eligible women were identified by midwives working at the MCHC, who passed on contact information to the researchers upon consent. The women were interviewed face-to-face, using a professional interpreter for most of the interviews. The interviews, lasting from 50 min to 1.5 h explored in detail the women’s experiences with maternity care in Norway, including potential barriers and facilitators. The included women received a reimbursement of 250 NOK for their participation – a gift card for use in a grocery store.

#### Qualitative part: in-depth interviews with midwives

In the qualitative part we additionally conducted in-depth interviews with seven midwives, three from hospitals and four from MCHCs in urban Oslo. The age of the midwives varied from 31 to 57 years. The interviews lasted between 1 and 2 h and included themes that focused on experiences and perceptions of maternity care with pregnant migrant women, challenges faced in their daily work and structural limitations related to time, resources and organisation of maternity care. We had initially planned 10 interviews with healthcare workers, however due to coronavirus pandemic, we had to pause the inclusion of the last 3 interviews. After starting analysis of the obtained material, data saturation had been reached, judged to be attained when no new themes or information emerged in subsequent interviews. We therefore decided to stop further data collection.

### Data analysis

The descriptive statistics from the quantitative data was analysed as mean with standard deviation (SD), median with interquartile range (IQR) and frequencies with percentage, using IBM SPSS version 25. The audio recorded in-depth interviews were transcribed and analysed using an inductive approach to identify recurring themes and sub-themes. The open-ended questions from the questionnaire and the qualitative data were analysed by thematic analysis. This involved reading and rereading the data, underlining key phrases and reoccurring topics and creating initial thematic codes. After reading the transcript, three researchers coded relevant sections separately, which were further discussed and modified if necessary. Themes and sub-themes were defined, and descriptive narrations were written and compared to the quantitative data material, drawing out quotes from migrant women and midwives that highlighted the four main themes identified in the transcribed interviews. In this article, the quotes from migrant women are followed by participant number, length of stay in Norway in whole years and reason for migration. For midwives, they are followed by number and workplace.

### Ethical considerations

The questionnaire study (approvals 18/15786 + 18/05310) and the in-depth interviews (approvals 18/15786) were approved by Oslo University Hospital and Akershus University Hospital’s ethical review committees. Information about the study was provided both orally and written to the migrant women and midwives. Written consent, or oral consent based upon the women’s preference, was obtained from those who volunteered to participate in the study. To ensure confidentiality, personal identification was removed, and all collected information including audio recordings, transcripts and questionnaires were securely stored and accessible only to the research team.

As the aim of this artice was on the barriers and challenges, we were conscious that participants reflections on these have the potential to reinforce negative ethnic or racial stereotypes as well as play into public discussions in media, especially on internet, on issues related to immigration, health-related deservingness and integration. Another important concern when conducting the in-depth interviews with pregnant migrant women was that participation may result in distress, or further trauma for those with a traumatic history. We made clear to the participants at the start of the interviews that they did not have to talk about issues they found difficult or too personal. If participants voluntarily shared traumatic issues, the research team informed participants of professional resources, including their midwives, for further support.

## Results

### Characteristics of migrant women

In the questionnaire study, 401 women participated, giving an 87.5% response rate. In total, the women were born in 66 different countries, with most belonging to the Central/Eastern European and Central Asian regions (Table 1). The five most frequent languages spoken at home were English, Polish, Arabic, Urdu and Tigrinya. For the in-depth interviews, 20 migrant women were included. The women were born in 12 different countries, with most belonging to the Sub-Saharan African region (Table 1). The languages Tigrinya, Arabic, Pashto, Sorani, Hindi, Portuguese, Russian and Uyghur were represented.

**Table 1:** Characteristics for recently migrated women from the questionnaire study and the in-depth interviews

Characteristics	Questionnaire study (*n* = 401)	In-depth interviews (*n* = 20)
**Mean age, in years (SD)**	29.8 (4.7)	30.1 (4.7)
**Mean length of stay, in months (SD)**	35.6 (19.4)	22.6 (14.2)
**Maternal region of birth, n (%)**		
Central/Eastern Europe and Central Asia	132 (32.9)	2 (10.0)
Latin America and Caribbean	13 (3.2)	1 (5.0)
North Africa and Middle East	76 (19.0)	5 (25.0)
South Asia	81 (20.2)	5 (25.0)
Southeast Asia, East Asia and Oceania	37 (9.2)	1 (5.0)
Sub-Saharan Africa	62 (15.5)	6 (30.0)
**Parity, n (%)**		
Primiparous	229 (57.1)	11 (55.0)
Multiparous	172 (42.9)	9 (45.0)
**Education, n (%)**		
No completed school	16 (4.0)	3 (15.0)
Primary/secondary school	151 (37.7)	8 (40.0)
University	234 (58.4)	9 (45.0)
**Reason for migration, n (%)**		
Refugee^a^	41 (10.2)	7 (35.0)
Family reunification	183 (45.6)	10 (50.0)
Education/work	177 (44.1)	3 (15.0)

^a^Refugee include undocumented migrants, asylum seekers and refugees

### Main barriers and challenges

**Fig. 1:**
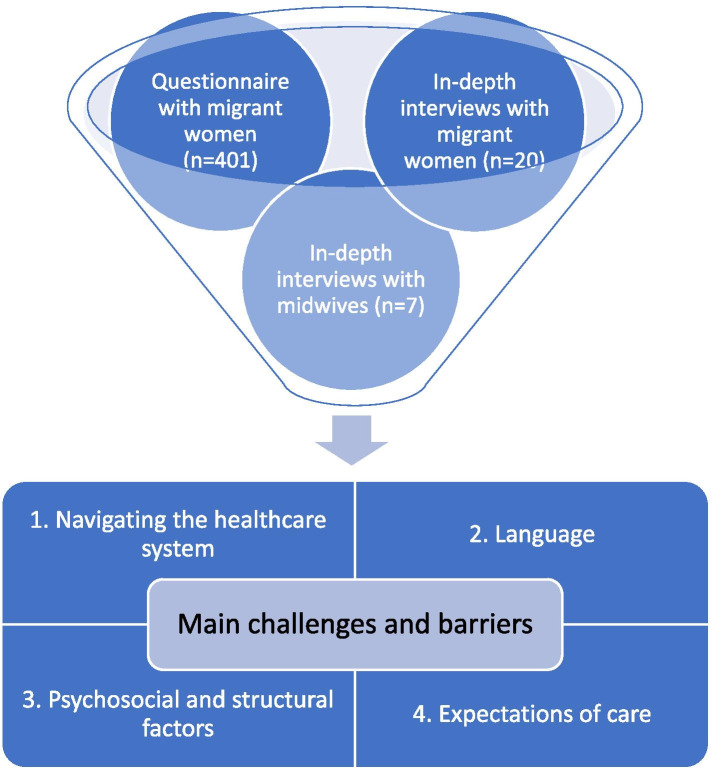
The main challenges and barriers identified by triangulating findings from structured questionnaire and in-depth interview with migrant women, and in-depth interview with healthcare personnel

Several challenges and barriers related to accessing and receiving care during pregnancy and birth in the questionnaire study and in-depth interviews were discussed. Combined, four main themes for challenges and barriers were identified: navigating the healthcare system, language, psychosocial and structural factors, and expectations of care (Fig. 1).

### Navigating the healthcare system

**Fig. 2:**
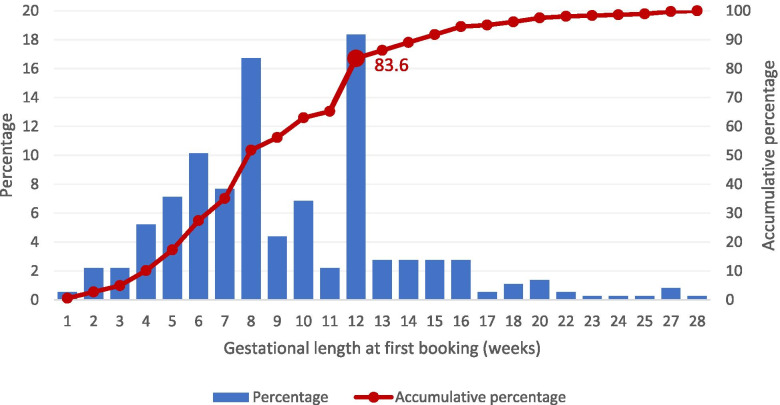
First antenatal booking by recently arrived migrant women from the questionnaire study, in percentage of all women (blue bars) and accumulative percentage (red line) by gestational length in weeks

*Navigating the healthcare system* was the most frequent barrier to receiving optimal healthcare, experienced by 185 women (46.1%) in the questionnaire study. Difficulties in navigating the health system included not realising that the services were offered, eligibility for those services and/or not understanding how the maternity healthcare system works. The median (IQR) time for booking the first antenatal care was 8 weeks (6 to 12), with 83.6% of the women having it done by week 12 (Fig. 2). Only 2.5% of the women had their booking after week 21. No significant difference was found for the women’s region of birth or migration background in terms of late antenatal booking (data not shown). The standard routine ultrasound conducted at around week 18 was attended by 93.5%. Early ultrasound, mainly done to detect health status or genetic characteristics of the foetus, which is currently not a part of routine antenatal care in Norway, was attended by 13.2%. Furthermore, less than one fifth (18.2%) had attended pregnancy courses through the MCHC or at the hospital prior to birth. During the study period, the pregnancy courses were only offered in Norwegian or, in very few places, English. Among the women who did not attend a course, 27.4% said they would attend a course if it was offered free of charge in a language they understood. Other services they would have liked to attend were courses about how the health system for maternity care is structured in Norway and a meeting place for pregnant women sharing the same language.

In the in-depth interviews, three sub-themes emerged: limited knowledge about the structure of healthcare system, long perceived waiting time for consultation and use of the emergency outpatient clinic. The majority of the women in the in-depth interviews stated low familiarity with the Norwegian healthcare system. Some had challenges with accessing appropriate healthcare due to lack of a personal identification number while others struggled to find information about their right to healthcare as foreigners in Norway. The Norwegian healthcare and welfare system is divided into different departments and this fragmented organisation can be especially difficult to navigate for recently arrived migrants. One woman described it this way:

*I was quite disappointed when I was followed up by my family doctor, because she didn’t give much information about how things happen in Norway…I have not lived here for long, she has to give some background.*

(Woman 9 - three years in Norway, education/work)

Explaining how the healthcare system is built, what rights the pregnant woman have for maternity leave and help in filling out forms for the Norwegian welfare system were common requests from migrant women to midwives. The midwives reported that newly arrived migrants struggled with a lack of familiarity with the Norwegian health and welfare systems, and their desire for orientation to accessible health services:

*Some people do not know anything about how things work here [in Norway] …they don’t know the system, for example how to apply for ultrasound, what they have a right to and can claim…there is a lot of information that must be conveyed [to the woman].*

(Midwife 1 - MCHC)

An undocumented woman explained how her first antenatal check-up was delayed due to lack of knowledge about available healthcare services, such as the Health Centre for Undocumented Migrants:

*I came to the health station [MCHC] very late because I did not know that I could get help there. My husband made inquiries, and since I was outside the system, they told us to get in touch with the health station and get help from them. In the beginning it was difficult since I did not have neither personal identification number nor a family doctor, and no one wanted to receive me.*

(Woman 10 - three years in Norway, undocumented migrant)

Late initiation of routine antenatal care, especially among undocumented migrants posed a challenge for some midwives, with time-consuming consultations and concerns about best care for the remaining pregnancy and birth:

*We had one here [undocumented woman] a while ago, she was in week 25, but never filled in a health card or applied for a birthing place [at a hospital].*

(Midwife 1 - MCHC)

Several migrant women described unfamiliarity with the process of booking a consultation for antenatal care and perceived prolonged waiting time at the family doctor:

*The system here is like you have to call to the family doctor and make an appointment…They give you time not on that same day…Maybe others have a [more] serious issue, you have not... But this is the bad thing, for me it's serious. So, you have to wait for two or three days*.

(Woman 1 - three years in Norway, family reunification)

When the women had acute concerns or symptoms, either related to the pregnancy, or other healthcare issues, many did not know whom to contact and ended up going to the Emergency outpatient clinic. As antenatal care is free of charge in Norway, some women were surprised when they had to pay for a consultation at the Emergency outpatient clinic:

*When I had to go to the emergency outpatient clinic, they gave me an invoice. My husband talked to them and told them that I was pregnant and therefore should not pay. They refused and said that we had to pay. We still haven’t paid that invoice, and now we have received warning of debt collection.*

(Woman 15 - two years in Norway, family reunification)

### Language

*Language* was the second most frequent barrier to receiving optimal healthcare, experienced by 112 women (27.9%) in the questionnaire study. Two-thirds (63.3%) of women would have understood the information during maternity care better if offered in another language. The Norwegian language proficiency among the migrant women was low; 22.9% of the women could not speak or understand Norwegian at all, 38.7% with difficulty and 38.4% had a good level. Almost one fifth of the women (17.2%) had contacted healthcare personnel in their country of birth for questions or concerns regarding their pregnancy and birth.

In the in-depth interviews, three sub-themes emerged: using a professional interpreter, anonymity and confidentiality, and use of relatives as interpreters. All migrant women mentioned language as an important barrier in accessing and using healthcare services, except those fluent in English. Even if they had relatively good Norwegian comprehension, there was a big gap between everyday language and medical terms, according to the women*.* Some women chose to have their antenatal care with their family doctor, as they had chosen a family doctor originating from the same country as themselves and therefore did not experience the language barrier. Corroborating the findings from the questionnaire study, some chose to speak to healthcare personnel in their country of birth, either digitally or even by visits to their country of birth for follow-up. Insufficient language proficiency was also one of the main challenges noted by healthcare personnel, that often lead to extended consultations to make sure they understood the concerns of the migrant woman or that the women understood the information provided by the healthcare personnel:

*We take them in for an extra consultation because there is so low language proficiency on the phone, things we would have clarified on the phone to people who spoke the language well, we have to take in to be sure...sometimes we almost do not understand what they are calling for.*

(Midwife 7 - hospital).

Challenges concerning use of interpreter was mentioned by many migrant women. Some women got an interpreter that spoke another dialect than they did and therefore encountered difficulties understanding the information:

*When I was new in Norway, I was in a car accident. I was in the hospital and there was an interpreter. I did not understand her dialect, so a big mistake happened, a big misunderstanding. The doctor wrote a lot of things I did not say, I even used a lawyer to change the statements, but they insisted that I said it.*

(Woman 2- five years in Norway, refugee).

Some migrant women were concerned about anonymity and confidentiality when using interpreting services. This was especially true for women who belonged to a community with a small number of people with the same ethnic background, and women who were suspicious of being under surveillance by authorities in their country of birth. One solution to language barriers and difficulties in getting a professional interpreter on time was using bilingual co-workers. Although midwives had good experiences with that, this option was not available for the majority of languages. Oftentimes the migrant woman’s relative or partners was used, however several midwives had concerns related to using relatives as interpreter:

*If you use relatives as interpreter, you do not really know how much they have understood. We do not really know what they are translating.*

(Midwife 2 - MCHC)

Discussing sensitive topics with relatives as interpreters or even a professional interpreter can be challenging, both for the patient and the healthcare personnel, as voiced by one midwife:

*If I know a woman comes in with a mother-in-law, I will not ask, for example, ‘how many induced abortions have you had? ´ But if there had been an interpreter and it was just her, I would have asked more easily about such things…and there may be sensitive things, so you do not necessarily want a woman to open up when there is an interpreter there.*

(Midwife 6 - hospital).

Among the English-speaking women a recurrent complaint was lack of English knowledge among the older healthcare personnel both at the MCHC and the hospital, as one migrant woman put it:

*I think that the old midwives, they don't like to speak in English...If you ask something, they always reply back in Norwegian. They understand…maybe they don't like that the new generation is speaking in English.*

(Woman 1 - three years in Norway, family reunification).

Although the Scandinavian languages Swedish and Danish are understood by most Norwegians, some migrant women emphasised that this is a challenge for migrants even though they have a fairly good command of the Norwegian language. One woman explained how she did not need an interpreter during her pregnancy, but when a Danish midwife attended her at the hospital for birth she did not understand much and was ashamed to ask for an interpreter, as it is expected to understand Scandinavian languages in Norway. In addition, while Norway has two official written languages, no spoken standard exists, making it hard for some migrants to understand the varying dialects in the country:

*People come from different regions and have different dialect in Norway. So even if you learn Norwegian in Oslo…if you speak to other people who come from other parts of Norway, it is difficult to understand that person.*

(Woman 9 - three years in Norway, education/work migrant)

### Psychosocial and structural factors

*Structural factors* were the third most frequent barrier to receiving optimal healthcare, experienced by 50 women (12.5%) in the questionnaire study. Structural factors included not having access to transportation, financial reasons, not getting time off work or not getting childcare for other children to attend services. Most women were married, while 21 women were single or divorced. Over 90% of the women lived with their partner, 22 women lived with their in-laws and 14 women lived alone. A bit more than half (57.3%) had paid work since moving to Norway, while 85.0% had work permit in Norway. Almost 20% answered that they experienced occasionally (15.0%) or often (4.7%) financial difficulties for the family the past year, for instance with making ends meet and paying monthly expenses such as food, often transportation and housing. In varying degrees, women reported symptoms of being afraid or anxious (24%), of hopelessness for the future (15%) and of loneliness (30%) (Table 2). Most of the women (96.8%) had someone they could trust, with whom they could speak in confidence and the partner was that person for the majority of the women (75.0%).

**Table 2:** The distribution of women from the questionnaire study (*n* = 401) who reported being troubled for three psychosocial symptoms, N (%)

Psychosocial symptoms	Afraid or anxious, N (%)	Sense of hopelessness for the future, N (%)	Sense of loneliness, N (%)
Not troubled	310 (77.3)	342 (85.3)	281 (70.1)
A little troubled	72 (18.0)	47 (11.7)	95 (23.7)
Very troubled	14 (4.7)	7 (1.7)	20 (5.0)
Extremely troubled	5 (1.2)	5 (1.2)	5 (1.2)

From the in-depth interviews, loneliness in the host country, distress about relatives in their country of birth and structural barriers emerged as sub-themes. Most of the women interviewed had a limited social network and many had close contact only with their in-laws:

*My husband has family here but as you know they've been living here for...So they are almost like Norwegians. Busy, busy, busy, busy, busy. You have to make an appointment first, then you have to ask them…So that's why I feel sometimes very lonely here because everyone is always busy.*

(Woman 1 – three years in Norway, family reunification)

Migrant women in general, and refugees especially, expressed distress about their relatives still in their country of birth and being under surveillance by the government:

*My brother is in jail now, because I'm abroad. They say that if I return to my homeland, they can give freedom to my brother. But that is not true. So I will not return, but I'm very sad about it. Every day I think about my brother and whether he is alive or not. Because I cannot have contact with him. My family too, we cannot talk on the phone*.

(Woman 3 – four years in Norway, family reunification)

Migrant women and midwives mentioned challenges beyond pregnancy and childbirth that to a great extent affected the migrant women’s lives. That included basic practicalities of everyday life, such as following up after consultations or reaching hospitals on time, as explained by a midwife:

*It gets too much [for the women]; if you speak the language poorly, not responsible for your own finances, do not have a driver's license… we say that ´you have to come now right away´, still it might take 3-4 hours, because they are waiting for the partner to come home from work and drive them. Or because they do not dare to come alone because they think they speak poor Norwegian. And many do not have the opportunity to leave their children at home, because they don’t know anyone who can be a babysitter.*

(Midwife 7 - hospital)

Even though maternity care is free of charge in Norway, certain deductibles may need to be paid which came as a surprise for some women. For instance, birth preparation courses are free of charge at some MCHCs while in other places it may cost a fee:

*It costs quite a lot to take those courses. At the hospital you pay 1300 NOK for two or three hours. There are not many districts that have it [for free], even though it is stated in the guidelines for maternity care that you must be able to offer birth preparation courses.*

(Midwife 1 - MCHC).

Another example of a financial challenge that midwives often observed among migrant women was related to transportation:

*We see many who want an ambulance to get in [to hospital], perhaps because they do not have a driver's license and they think it is too expensive with taxi. It also becomes a problem to explain, that we think it is acute enough that they should come to check-up, but not so acute that they need ambulance transport. Then they may choose not to come for the check-up, because they have to pay NOK 500 in a taxi to enter.*

(Midwife 7 - hospital)

Both migrant women and midwives addressed how legal restrictions and lack of a residence permit made the migrant women’s life more complicated. After moving to Norway, one woman had to leave her two children in Norway because of a forced return to her country of birth:

*I lived in my home country for one year and seven months without my husband, daughter [2 years old] and son [4 years old], it was really hard.*

(Woman 15 - two years in Norway, family reunification)

One midwife explained how an undocumented pregnant migrant woman faced several problems beyond the pregnancy:

*She had experienced a lot of violence, did not have a place to live and in addition great challenges in relation to health.*

(Midwife 1 - MCHC)

### Expectations of care

Seventeen women experienced that healthcare personnel refused a practice or ritual during or after birth that she requested, in the questionnaire study. Some of these wishes were related to food preferences. One woman asked to pierce her infant’s ears as per cultural custom, but was refused by health personnel for fear of causing pain to the child. Other women requested bathing the infant right after birth, which was rejected by health personnel because it was not standard Norwegian custom. Another woman wanted to perform an ‘adhan,’ a traditional Islamic birth custom, but was rejected for concerns of impairing the infants’ hearing. Six women reported that they wanted to bring more relatives or support persons into the birthing room than was allowed.

From the in-depth interviews, conflicting recommendations, varying support from family and gender preference on healthcare personnel emerged as sub-themes. Differences in recommendations for physical activity in pregnancy and after birth was a repeating topic of discussion by both migrant women and midwives. Migrant women reported conflicting advice on how much physical activity was beneficial during pregnancy. One woman explained how her relatives residing in her country of birth reacted to the recommendations for physical activity during pregnancy and after birth in Norway:

*When I told them [relatives from home country], they reacted by saying that I was completely crazy and had lost my mind, and that it was crazy to go out after only a week!*

(Woman 10 - three years in Norway, undocumented migrant).

Midwives explained how the difference in their recommendations about level of physical activity after birth and some women’s own expectations and experience from their birth country could lead the midwives to view the migrant women as lazy and less co-operative. Eventually, this could make patient-provider relationships more challenging as well as have the potential to contribute to cementing attitudes and cultural stereotypes about the women. As one midwife noted:

*Sometimes it's hard to get them up. Especially after a caesarean section…they may think we're mean or want to punish them...What is a pity are attitudes among staff in the department, it often becomes like ‘she is so hard to get up, ‘she wants nothing’, but that’s often not what it’s about. It's more about the fact that they haven’t understood why they should do it*.

(Midwife 4 - hospital).

Both migrant women and midwives observed a cultural difference in how much help the pregnant women got from relatives. Perceived increased responsibility for the newborn and individualistic lifestyle in the host country was a transition for some migrants:

*When you give birth in Norway, you have a responsibility to the child, the home and everything else…In my home country it is very different, there your mother comes and is with you for a whole month and other relatives help. It is almost as if you do not notice that you have a child.*

(Woman 10 - three years in Norway, undocumented migrant).

Bringing many relatives to the hospital when giving birth and post-partum was a recurring difference in expectations between migrants and the majority population. One midwife explained how this practice was perceived as unfamiliar to the midwives, yet not allowing visits could contribute to feelings of isolation in migrant women:

*When they bring with them maybe five, six, seven, eight, ten, people, from the start till birth, which can take three days, then we feel that it is different than what we are used to at the ward. I think I forget to think that this is perhaps what the woman is used to from before and needs to feel safe, if we send home all the people, it will suddenly be a very insecure situation.*

(Midwife 7 - hospital).

The midwives had experienced some incidents where the migrant woman did not want a male healthcare personnel. A few women emphasised the importance of having female healthcare personnel, mostly for clinical work and check-ups, but also for having a female interpreter:

*I have told the family doctor that I need a female interpreter, but they say that they don’t have female interpreters, and I don’t want a male interpreter… at the family doctor there is someone who speaks Arabic. There is a man, so despite the fact that I have said several times that I do not want a male interpreter, he still comes and interprets.*

(Woman 15 - two years in Norway, family reunification).

## Discussion

This article investigated potential barriers and challenges to optimal maternity care for recently arrived migrants as perceived by the migrant women and midwives. The challenges they reported as most difficult were related to navigating the healthcare system, language, psychosocial and structural factors, and expectations of care. Even though our findings are consistent with previous international literature on perceived barriers among migrant women, until now few studies have explored barriers in particular for *recently* migrated women. Lack of knowledge about the healthcare structure and limited social network during the first period after having migrated to the country emerged as significant challenges for the recently migrated women.

The healthcare services in Norway are comparably of a high standard [25]. The fact that the accessibility and quality have been so high over many years, may also contribute to higher expectations of its service delivery, and potentially a lower threshold for criticising the health system and its services. Yet, our findings do suggest that some migrant women had variable layers of vulnerability factors that influenced their capacity and means to use the health services available and to understand and navigate the health system.

In agreement with previous studies, we found that migrant women lacked information about the healthcare system in host countries, including administrative procedures, which led to women not using the variety of available maternity care services [9, 17, 26]. National guidelines in Norway recommends the first antenatal care consultation to be booked by the end of gestational week 12 [20], which was done by 83.6% in our study. As we did not compare migrants to non-migrants, we cannot establish if there was a difference in how early the women started antenatal care. Nevertheless, studies from European countries have shown later initiation of antenatal care among migrants compared to non-migrants [27, 28], first generation- compared to second generation migrants [29], minority ethnic groups compared to White women [28, 30] and especially profound among recently migrated women [31]. Although our finding of a high percentage of timely initiation of antenatal care, midwives from the in-depth interviews indicate that subgroups of migrants may be at risk. Our findings should therefore be further explored by research on subgroups with low language proficiency, acculturation and among undocumented migrants [13].

Slightly lower attendance was found for the standard routine ultrasound conducted at around week 18, which was 93.5% in our study, compared to 97% in national surveys [32] . The high attendance for standard routine ultrasound in our study may be explained by the relatively high number of women from Central and Eastern Europe that were included, seeing that there is a practice and expectation of using ultrasound earlier and more frequently during pregnancies in those countries [33]. We also found that 13.2% of the women had gotten an early ultrasound, a service often paid for privately as it is not a part of routine antenatal care in Norway, except for groups with elevated risk of fetal chromosomal abnormality. This is low compared to local surveys in Norway suggesting that half of the women had an early ultrasound in the first trimester [32]. Women reported often using the emergency outpatient clinic in case of medical concerns, in line with a previous study that found more frequent use of emergency outpatient clinic by migrants compared to the host population [34]. Educating the migrant women about the structure of healthcare system may be a solution in reducing the barriers of navigating the healthcare system.

Our findings on language barriers, complements previous work where language is highlighted as one of the main barriers for migrants [1, 15–17]. Use of interpreter services have been shown to increase the understanding of maternal health information among migrants [35]. However, we found that even when a professional interpreter was used, sometimes communication problems persisted as a result of dialect or gender of interpreter. Healthcare personnel, as well as the institutions they are part of, need to be aware of this and the need for appropriate interpretation services. Furthermore, previous research has linked low language proficiency to low attendance in pregnancy preparation courses among migrants [36, 37]. Therefore, offering pregnancy preparation courses in English and other major languages could be beneficial in increasing the attendance among non-Norwegian speaking women.

Our findings show that recently migrated women often lacked social support, had limited social network and struggled to acclimate to the difference in community and familial support between their birth country and Norway. Previous studies on social support among migrants are not conclusive, as some are in concordance with our findings [30], while others found no evidence of limited social support [8], or even higher social support in migrant groups [8, 38]. Longer length of stay in the host country often leads to wider social networks. This could explain why the recently arrived women in our study experienced limited social networks as challenging – psychosocially as well as in relation to practical and emotional support. Lack of social support has been shown to be linked with a number of adverse pregnancy outcomes, such as post-partum depression [39, 40], low birth weight [41] and preterm birth [42]. Identifying women that lack or have little social support and providing them with additional social services may thus increase psychosocial wellbeing as well as potentially identify additional vulnerability factors.

Varying expectations of care and the healthcare system’s limited ability to provide differentiated care to women with special needs, may make it difficult for migrant women to adjust to the healthcare system in host countries [14]. While coping with conflicting recommendations in the two countries, migrant women can even be viewed as “*difficult to manage*” by healthcare personnel. Although some training in cultural competence is offered during professional education, efforts to include more targeted training for health personnel, both during professional education but also as continued learning could provide increased awareness and self-reflexivity. As explained by Phillimore et al. [26], it is almost impossible to gain cultural knowledge about every ethnic group in an increasingly multi-ethnic world. Rather, focus should be on intercultural competence and treating patients individually while still being culturally sensitive. A newly published scoping review on different models of antenatal care targeted at migrant women, including group antenatal care and specialised clinics, found the models to be acceptable for women and increased access to care [43]. Use of multicultural doulas for vulnerable migrant women have shown promising results in Norway [44].

This article has not explored conceptions of ‘health related deservingness’ [45] – who ‘deserves’ or have the right to access health services or who should or should not be financially supported when accessing services. The question of who deserves it most and the extent to which diverse migrant groups can claim state welfare goods is often debated in Norwegian media and on internet sites. The competing and black-and-white stances are often grounded in moral judgement, notions of exclusive citizen rights, and moral ideas about having to ‘earn’ access to goods. The extent to which these contentions and judgments find their way into healthcare provision in Norway needs further exploration.

### Strengths and limitations

Strengths of this study include an emphasis on multidisciplinary research, from the design phase to interpretation of findings, as the authors hold background in medicine, gynaecology, anthropology and public health. Two authors, one physician and one medical anthropologist, performed the content analysis independently and discussed the findings before reaching consensus, thereby increasing the validity. Both the questionnaire study and the in-depth interviews were done face-to-face in the migrant women’s language of choice, enabling women with low language proficiency and literacy to participate. A high response-rate for the questionnaire study with few missing values limited response bias. The in-depth interviews were conducted by anthropologists, limiting the possible social desirability bias that using healthcare personnel can introduce.

Nonetheless, limitations exist. Administering the questionnaire-study within some days of birth could potentially introduce bias as the new mothers might be exhausted and not remember details about the pregnancy well. This timing, however, ensured responses from hard-to-reach groups, a factor we considered more important. As healthcare personnel conducted the quantitative interviews, social desirability bias could affect the answers of the migrant women. Limitations of the in-depth interviews include convenience sampling and selection bias. With midwives at the MCHCs holding responsibility for recruiting eligible migrant women, the women interviewed might represent a group of migrants who are more integrated, omitting those who were most isolated and did not attend MCHCs. The findings reported from the in-depth interviews with midwives are based on purposive sampling of healthcare personnel who volunteered to participate in the study. Therefore, the extent to which the midwife’s views are representative of all healthcare personnel serving migrant women is unknown. In addition, taking the sample only from a diverse urban area may limit the generalisability of the findings in rural areas.

We did not explicitly focus on gender relations and to what extent cultural understanding of gender influence access to maternal healthcare services. Issues related to not reaching hospital in time when experiencing symptoms, for example due to lack of childcare or transportation, may reflect gendered divisions of responsibilities or culturally shaped notions of birth belonging to the ‘women’s sphere’. Furthermore, the fact that all participants included in our study were women, men’s voices and perceptions have not been included, and thus gendered norms and the ways they may influence uptake of services have not been explored.

## Conclusion

Low familiarity with the healthcare system in the host country can hinder recently arrived migrant women in navigating and utilising the maternity services. Combined with, limited language proficiency, psychosocial/structural factors and different expectation of care, they are the main challenges and barriers to optimal maternity care for migrant women. Improvements and interventions that may meet the needs of the recently arrived migrants include improved provision of health system structure, appropriate use of professional interpreter, broader range of social services offered to women with limited social network and increased cultural competency among healthcare personnel.

## Supplementary Information

**Additional file 1.**

## Data Availability

The datasets generated and analysed during the current study are not publicly available due to protection of individual participants’ privacy and confidentiality, but are available from the corresponding author on reasonable request.

## Abbreviation

MCHC: Maternal and Child Health Centre
